# Incidence and Risk Factors Associated With Hospitalization for Variant Angina in Korea

**DOI:** 10.1097/MD.0000000000003237

**Published:** 2016-04-01

**Authors:** Hack-Lyoung Kim, Sang Hyung Lee, Jayeun Kim, Hyun Joo Kim, Woo-Hyun Lim, Jae-Bin Seo, Woo-Young Chung, Sang-Hyun Kim, Joo-Hee Zo, Myung-A Kim, Jin Yong Lee

**Affiliations:** From the Division of Cardiology, Boramae Medical Center, Seoul National University College of Medicine (H-LK, W-HL, J-BS, W-YC, S-HK, J-HZ, M-AK); Public Health Medical Service, Boramae Medical Center, Seoul National University College of Medicine (SHL, JYL); Department of Neurosurgery, Seoul National University College of Medicine (SHL); Institute of Health and Environment, Seoul National University (JK); Department of Nursing Science, Shinsung University (HJK); and Institute of Health Policy and Management, Medical Research Center, Seoul National University (JYL), Seoul, Korea.

## Abstract

This study aimed to determine the incidence and the risk factors of hospitalization for variant angina (VA) in Korean patients. Using the National Inpatient Sample (NIS) database, manufactured and released by the Health Insurance Review and Assessment Service (HIRA) in Korea, the incidence of hospitalization and rehospitalization for VA were calculated. The numbers of patients hospitalized for VA were estimated to be 14,362 in 2009, 17,492 in 2010, and 20,592 in 2011. The standardized incidence rates of hospitalization for VA were 31.4% in 2009, 36.5% in 2010, and 41.7% in 2011 (relative increase rate from 2009 to 2011, 33.0%, *P* for trend < 0.0001). VA patients predominantly belonged to the middle-age group between 40 and 69 years (75.5%), and there were 54.3% male. Based on the hospitalization episodes, the number of rehospitalization was calculated to be 879, 1141, and 1446 patients out of 1867, 2274, and 2677 patients from 2009, 2010, and 2011, respectively. The rates of rehospitalization for VA were 47.1% in 2009, 50.2% in 2010, and 54.0% in 2011 (*P* for trend < 0.0001). Age was an independent factor associated with rehospitalization for VA. Hospitalization for VA occurred most frequently in fall from 2009 to 2011. In conclusion, hospitalization rates for VA steadily increased from 2009 to 2011 in Korea, and about a half of VA patients was hospitalized more than once a year in 2009 to 2011. Proper health policy and patient education are warranted to control the high rate of hospitalization for VA.

## INTRODUCTION

Variant angina (VA) is caused by reversible coronary artery spasm, which is characterized by chest pain with ST segment elevations on electrocardiogram at rest, and is not induced by exercise in the daytime.^1^ The prognosis of VA is relatively benign as long as patients are on vasodilator therapy, and avoid smoking and alcohol drinking.^1,2^ VA mostly affects relatively healthy and young people who are very productive and actively involved in social activities. However, VA attack often requires hospitalization and restricts social activities of such people. In particular, fatal complications, such as acute myocardial infarction, ventricular arrhythmia, and sudden cardiac death, can develop during severe coronary vasospasm.^3,4^ Therefore, in these cases, VA can result in catastrophic disease burdens not only in individuals but also in their families and societies.

In spite of this clinical importance of VA, little is known about the incidence of VA, except that it is substantially less common than typical angina caused by atherosclerotic coronary stenosis.^5^ Even though some studies revealed the prevalence of VA in some Western countries and Japan^6–9^ and several studies have reported that VA is highly prevalent in Korea^10–13^; these studies have limitations in terms of generalization due to their small sample size or single-center design. Therefore, it is difficult to obtain the local or global level of epidemiological information of VA.

To understand a disease, we need to investigate its clinical manifestations, pathophysiology, contributing factors, diagnostic methods, natural history and prognosis, treatments, and complications. The first thing we must do is to grasp the whole scale of disease in order to gather and summarize these kinds of clinical information. Therefore, we should know epidemiological information as to how many disease cases newly occur during a specific period, and whether there is any specific pattern of the disease, such as seasonal variation.

In Korean patients with VA, this study was conducted to determine the incidence of hospitalization for VA, to confirm whether hospitalization for VA shows seasonal variations, and to explore factors affecting the incidence of rehospitalization for VA.

## METHODS

### Data Source

We used the National Inpatient Sample (NIS) database from 2009 to 2011, which is annually manufactured and released by the Health Insurance Review and Assessment Service (HIRA) in Korea. The more detailed information regarding the NIS can be found in the studies of Kim et al^14^ and Lee et al.^15^ In short, the NIS is a sampling data set, which consisted of 1% of outpatients and 13% of inpatients extracted from the whole Korean population. In 2011, the NIS contained 765,564 inpatient samples actually representing 6,026,063 of the total inpatients in Korea. Each of the claims or patients in the database is designed to represent 100 claims or 100 patients.

### Operational Definition

#### Patients with VA

Patients who were hospitalized for the primary diagnosis code of I201, based on International Classification of Diseases-10th Revision.

#### Annual Incidence

The number of inpatients who were newly diagnosed with VA (I201, the primary diagnostic code) in 2009, 2010, and 2011, respectively.

#### Rehospitalization

The number of inpatients who were hospitalized more than twice for the primary diagnostic code of I201 in 2009, 2010, and 2011.

#### Season

Seasons were defined as spring (March–May), summer (June–August), fall (September–November), and winter (December–February) at first hospitalization of each patient.

### Calculation of Incidence of Hospitalization and Rehospitalization for VA

From the NIS 2009, 2010, and 2011, we extracted each annual admission cases hospitalized for VA by searching the primary diagnosis code of I201 with admission record. A total of 6818 patients in 2009 to 2011 (1867, 2274, and 2677 patients, respectively) were finally searched, which were imputed into the Korean population size using sampling weight. The numbers of patients hospitalized for VA were estimated to be 14,362 in 2009, 17,492 in 2010, and 20,592 in 2011. These numbers represent crude incidence of hospitalization for VA. We also yielded annual standardized incidence rates (SIRs) after adjusting for sex and age. Finally, the SIRs were computed as the annual inpatient cases per 100,000 people.

In cases of rehospitalization in a specific year, we only kept the earliest (oldest) claim data because the NIS is a sampling data per year and also because only annually framed data were available to figure out rehospitalization. Based on the hospitalization episodes, the number of rehospitalization was calculated to be 879, 1141, and 1446 patients out of 1867, 2274, and 2677 patients from 2009, 2010, and 2011, respectively.

### Statistical Analysis

Incidence rates of hospitalization for VA were calculated in 2009, 2010, and 2011, according to sex, age group, type of health insurance, residence area, co-morbidity, and month/season of hospitalization. In the analyses, patients were divided into 10-year age groups. Pearson chi-squared test was used to examine the difference in characteristics of VA in a specific year, and the Cochran–Armitage trend test to detect the increasing or decreasing trend of the patterns of VA between age groups during 2009 to 2011. Then, multivariable logistic regression was performed to examine the association between hospitalization and demographic characteristics, such as sex, age, and type of insurance and medical center. All the analyses were completed using SAS, version 9.3 (SAS Institute, Inc., Cary, NC) and all statistical tests were 2-sided, and a *P* value of <0.05 was considered statistically significant.

### Ethical Statement

This study was exempted from the approval by the Institutional Review Board of Seoul National University Boramae Medical Center (Seoul, Korea, IRB No. 07-2015-1). Informed consent was not obtained because patient records/information was anonymized and deidentified prior to analysis.

## RESULTS

**FIGURE 1:**
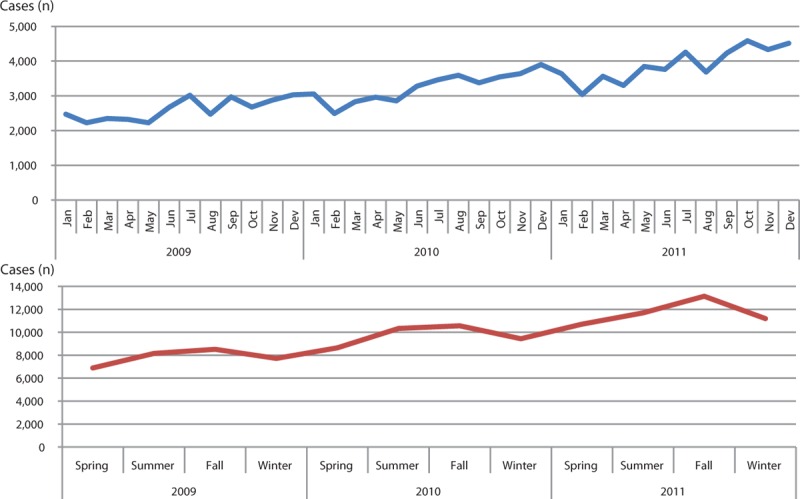
Time series distribution of hospitalization for variant angina from 2009 to 2011 (based on the episodes of hospitalization).

**FIGURE 2:**
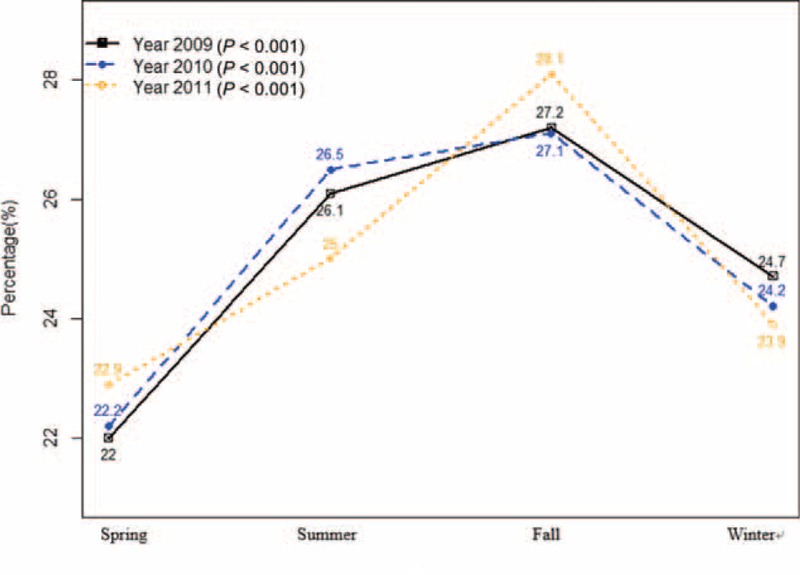
Seasonal variation in hospitalization for variant angina from 2009 to 2011.

The characteristics of hospitalization for VA are shown in Table 1. The SIRs of VA hospitalization were 31.40 in 2009, 36.59 in 2010, and 41.77 in 2011 (relative increase rate from 2009 to 2011, 33.0%, *P* for trend < 0.0001). VA patients predominantly belonged to the middle-age group between 40 and 69 years (75.5%), and there were 54.3% male. Most of the VA patients had health insurance (92.4%) and were admitted to a tertiary teaching hospital (45.2%) and a general hospital (48.9%). The characteristics of patients with a history of rehospitalization for VA are presented in Table 2. About half of patients with VA were hospitalized more than once a year. The incidence rates of rehospitalization for VA were 47.1% in 2009, 50.2% in 2010, and 54.0% in 2011 (*P* for trend < 0.0001). Rehospitalized patients predominantly belonged to the middle-age group between 40 and 69 years (77.9%) and there were 54.1% male. The proportions of patients with health insurance were 90.6% in 2009, 91.7% in 2010, and 92.3% in 2011 (*P* for trend = 0.0006). Factors associated with rehospitalization for VA are shown in Table 3. There were no gender differences in rehospitalization incidence rates between years 2009, 2010, and 2011 (*P* > 0.05). Old patients were at higher risk of rehospitalization compared to young patients. Medical aid was associated with rehospitalization in 2009, but not in 2010 or 2011. There were significant seasonal variations in hospitalization for VA (Figures 1 and 2). VA hospitalization was most frequently noted in fall during 2009 to 2011. There was a 5.1% difference in hospitalization for VA between fall and spring (27.4% vs 22.3%).

**TABLE 1:**
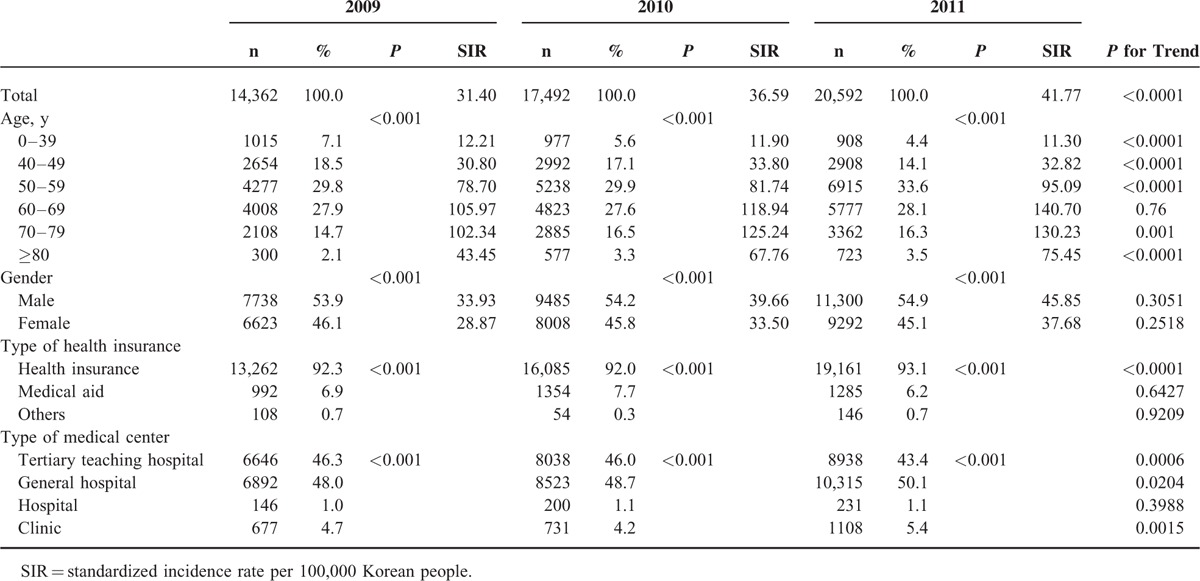
Characteristics of Variant Angina Hospitalization in Korean Population Between 2009 and 2011

**TABLE 2:**
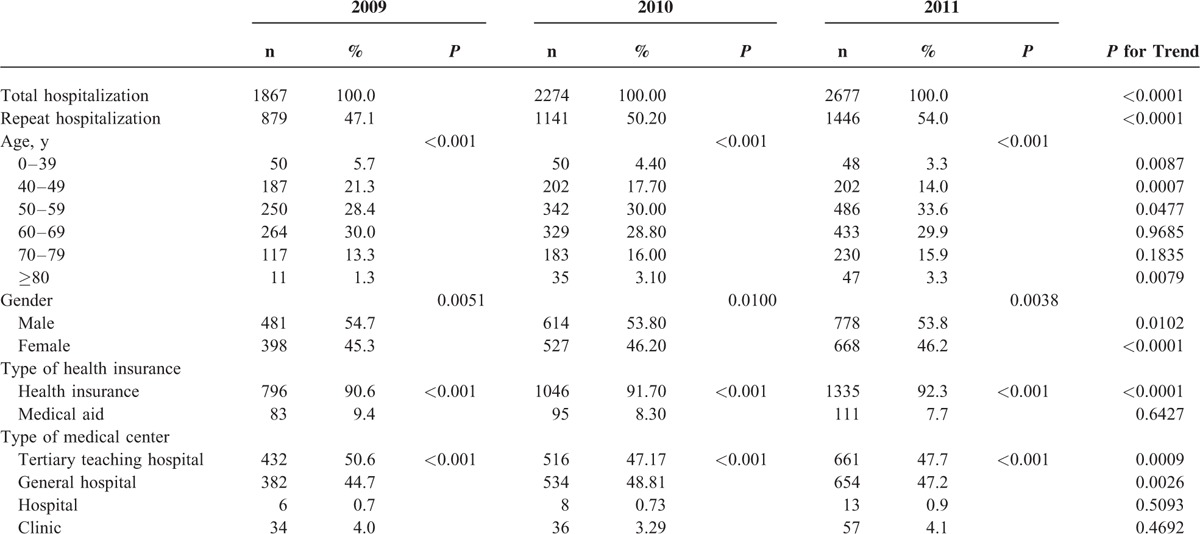
Characteristics of Patients With Repeat Hospitalization for Variant Angina in Korean Population Between 2009 and 2011

**TABLE 3:**
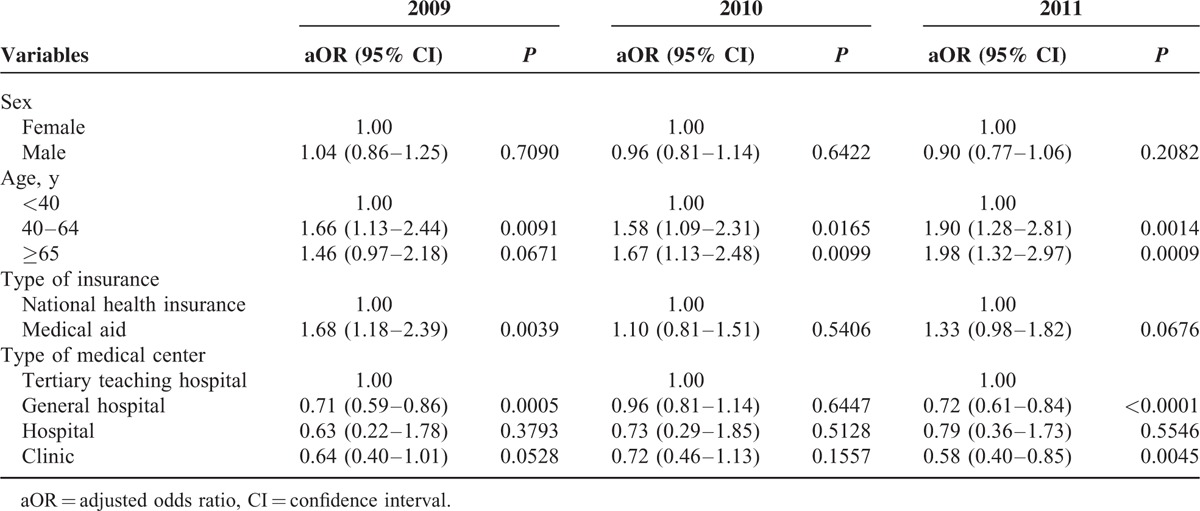
Factors Associated With Repeated Hospitalization due to Variant Angina

## DISCUSSION

This study using the nation-wide database demonstrated several important findings: there was a significant increase in hospitalizations for VA during the years of 2009 to 2011 in Korea; most of the hospitalizations were noted with middle-age group between 40 and 69 years; rehospitalization for VA increased continuously during the study period; old age was an independent factor associated with rehospitalization for VA; and there was a significant seasonal variation in hospitalization for VA, which were more frequent in summer and fall than in spring and winter.

### Incidence of VA Hospitalization

To the best of our knowledge, there has been lack of reports on the incidence of hospitalization for VA. Our results firstly showed that the incidence of hospitalization for VA in Korean subjects. We also calculated the SIR of VA hospitalization. This may be a valuable data for researchers, and clinicians and the development of healthcare policy. More important, a gradual increase in the incidence of hospitalization and rehospitalization for VA suggests a simultaneous increase in health costs for the treatment of VA in Korea. Moreover, the fact that most of the hospitalized patients were at working age has clinical and political implications. Loss of the labor potential in these young people is always a burden to the social and economic welfare of a society as well as a loss of important life time for individuals and their families. Considering that VA can be well controlled by regular medications and avoiding smoking and alcohol consumption, patient education is warranted to control high incidence rates of hospitalization for VA.

### Seasonal Variations in the Incidence of VA

Although circadian variations in VA attack have been well described,^16,17^ there has been limited data as to whether there are seasonal variations in hospitalization for VA. Therefore, seasonal variations have rarely been taken into account in the clinical management of VA so far. Our study identified a significantly increased incidence of hospitalization for VA in fall than in the other seasons. Unlike myocardial infarction, stroke, and heart failure, the incidence of hospitalization for VA was not associated with cold weather. Although the main underlying mechanism for this finding has not yet been completely elucidated, a plausible hypothesis is that variations in daily temperature may more frequently provoke vasospasm in fall. Our results suggest that sudden changes in temperature more frequently induce coronary vasospasm than cold temperature itself. In addition, seasonal variations in the incidence of hospitalization for VA appear to reflect variations in lifestyle risk factors such as cigarette smoking and alcohol drinking. Moreover, given that allergens in the air are abundant during fall in Korea, allergic reactions can also be suggested as a possible pathophysiologic mechanism explaining seasonal attacks of VA.^18^ Previous studies have demonstrated that there is the relationship between allergic reactions and coronary spasm, and that the recurrence rate of VA can be reduced by treatment with steroids or antihistamines.^19–22^ Vasoactive peptides released from circulatory or cardiac mast cells through the allergic process may cause coronary spasm.^23,24^ Further studies are needed to confirm our results.

### Factors Associated With Repeated Hospitalization for VA

Our results are the first to show the annual incidence rate of rehospitalization for VA. Although we did not compare our data to previous ones, our data unexpectedly showed that about half of VA patients were hospitalized more than once a year. The high incidence rate of rehospitalization for VA, even after effective medical treatments such as vasodilators, suggests need for more medical attention on patients with VA. Identification of risk factors associated with rehospitalization is important in the management of VA patients, and the establishment of a proper health policy. Therefore, we attempted to find out the risk factors for rehospitalization for VA; however, there were no significant risk factors except age.

### Political Implications for Health Authorities

VA is one of the ambulatory care-sensitive conditions, whose hospitalization can be prevented by utilizing primary appropriately utilizing primary care. Hospitalization for VA could be reduced or minimized by providing high-quality outpatient care.^25–27^ However, our results showed that the incidence of hospitalization and rehospitalization for VA during 2009 to 2011 continuously increased. This result may be interpreted to mean that the primary care system in Korea is not appropriately being functioned.^15^ Therefore, health authorities should pay more attention to whether there is any problem of healthcare delivery systems (i.e., the cooperation between primary care and referral hospitals).

### Study Limitations

Besides retrospective analysis, our study has several limitations. First, errors from possible coding inaccuracy may be the main limitation of such administrative databases. Second, given that the data were mainly from the primary diagnoses at discharge, there was a possibility that the incidence hospitalization for VA was underestimated if VA was accompanied by more serious clinical conditions such as myocardial infarction or sudden cardiac death. Our study focused on hospitalizations for VA and did not include any data pertaining to VA care in outpatient clinics or emergency departments. Lastly, information regarding VA complication, mortality, long-term follow-up outcomes, medications, and medical cost in the study patients was lacking. Such information could be valuable in proper healthcare planning and management. In spite of these limitations, the present study has clinical implications because a real-world large sample size with national database was used, and also because there was no selection bias.

## CONCLUSIONS

Hospitalization for VA continuously increased from 2009 to 2011 in Korea, and about half of VA patients were hospitalized more than once a year during this period. Ongoing effort with proper health policy and patients education are warranted to control high rates of hospitalization for VA.
