# Incidence and Risk Factors Associated With Hospitalization for Variant Angina in Korea: Erratum

**DOI:** 10.1097/01.md.0000484986.12820.2e

**Published:** 2016-06-24

In the article “Incidence and Risk Factors Associated With Hospitalization for Variant Angina in Korea”,^1^ which appeared in Volume 95, Issue 13 of *Medicine*, a sentence in the abstract has been replaced. The correct sentence reads “The standardized incidence rates of hospitalization for VA were 31.4 per 100,000 people in 2009, 36.5 in 2010, and 41.7 in 2011 (relative increase rate from 2009 to 2011, 33.0%, *P* for trend < 0.0001). The article has since been corrected online.
